# A review on proteomic and genomic biomarkers for gelatin source authentication: Challenges and future outlook

**DOI:** 10.1016/j.heliyon.2023.e16621

**Published:** 2023-05-24

**Authors:** Marco Garcia-Vaquero, Armin Mirzapour-Kouhdasht

**Affiliations:** School of Agriculture and Food Science, University College Dublin, Belfield, Dublin 4, Ireland

**Keywords:** Proteomic, Genomic, Biomarker, Gelatin, Halal, Kosher

## Abstract

Biomarkers are compounds that could be detected and used as indicators of normal and/or abnormal functioning of different biological systems, including animal tissues and food matrices. Gelatin products of animal origin, mainly bovine and porcine, are currently under scrutiny mainly due to the specific needs of some sectors of the population related to religious beliefs and their dietary prohibitions, as well as some potential health threats associated with these products. Thus, manufacturers are currently in need of a reliable, convenient, and easy procedure to discern and authenticate the origin of animal-based gelatins (bovine, porcine, chicken, or fish). This work aims to review current advances in the creation of reliable gelatin biomarkers for food authentication purposes based on proteomic and DNA biomarkers that could be applied in the food sector. Overall, the presence of specific proteins and peptides in gelatin can be chemically analysed (i.e., by chromatography, mass spectroscopy, electrophoresis, lateral flow devices, and enzyme-linked immunosorbent assay), and different polymerase chain reaction (PCR) methods have been applied for the detection of nucleic acid substances in gelatin. Altogether, despite the fact that numerous methods are currently being developed for the purpose of detecting gelatin biomarkers, their widespread application is highly dependent on the cost of the equipment and reagents as well as the ease of use of the various methods. Combining different methods and approaches targeting multiple biomarkers may be key for manufacturers to achieve reliable authentication of gelatin's origin.

## 1 Introduction

Biomarkers used for food origin authentication purposes are molecules which presence relates to a specific species. Two prominent categories of these molecules are proteomic (proteins and peptides) and genomic. To be considered a proper biomarker, these molecules should ideally be responsive, specific, and applicable [1].

Collagen, a fibrous protein abundant in different animal tissues like skin, bones, and connective tissue, can be partially hydrolysed to extract gelatin, which is extensively used in food and pharmaceutical applications [[2], [3], [4], [5]]. There are two unique types of gelatin, namely types A and B, resulting from the pH of the pre-treatments used to generate these compounds. Type A gelatin is produced by using acid pre-treatments, and it has an isoelectric point at pHs 6–9, whereas type B gelatin, generated by alkaline pre-treatments, has an isoelectric point at pHs 4.8–5.4 [6]. Porcine and bovine skins and bones are currently the most commonly utilized materials worldwide to supply the demands of gelatin for multiple applications [7,8]. However, there are currently public health concerns related to their use as they may be a source of pathogen dissemination, such as prions associated with the consumption of mammalian gelatins [9,10]. Moreover, the religious beliefs of some sectors of the population are a limiting factor in the use of gelatins of animal origin by consumers. The dietary laws and requirements followed by Muslims (Halal food) and Jews (Kosher food) need to ensure that food products are free from porcine products and by-products, while Hindu customers demand food products of no bovine origin [3,11,12]. On the other hand, gelatin is also utilized as a supplement or traditional medicine, such as donkey-hide gelatin, a traditional Chinese medicine manufactured from donkey skin [13,14].

Recognizing the source of gelatin is also important for the consumers of this high-value product in order to avoid product fraud. For instance, although industrial gelatin made from leather waste can have a significant level of chromium, which can lead to kidney damage and, in extreme situations, be linked to cancer [15], some retailers have substituted industrial gelatin for edible gelatin in food products due the low cost and ease of manufacture of this product. Sausages and pork jelly are food products that are widely appreciated as a delicacy in numerous regions of China, and both items contain gelatin. The marketing expenditure allocated to the promotion of these food products exceeds $15 million [16]. Furthermore, other cases of food fraud were reported by Demirhan, Ulca [17] that discovered porcine DNA in some Halal food products, such as marshmallows, gum drops, and Turkish delights, that were declared to be made with Halal gelatin when they were tested. Due to worries about allergies, law, ethics, and the practice of certain religions, using less expensive materials in edible bird's nests, such as porcine gelatin, can cause issues. Using a mix of chemometrics analysis and Fourier transform infrared spectroscopy (FTIR), porcine gelatin was found in edible bird's nests in one instance. Porcine gelatin was discovered through PCA analysis of FTIR data, indicating at least 5% adulteration in edible bird's nests [18].

On the basis of these consumer demands and increased public health concerns, researchers are working on the most reliable and convenient approaches for manufacturers to allow the identification of the original sources of gelatins. Several methods have been established, although there is not an officially authentication method defined yet, as each method has its own advantages and limitations in its implementation. On the other hand, even though some effective methods have been successfully developed in many countries, the excessive prices of equipment/reagents of some of these techniques do not allow their regular and widespread use. One of the highly acceptable strategies is the application of proteomic biomarkers, including proteins and peptides, that could be used to determine the original source of gelatins [[19], [20], [21], [22], [23]], while genomic-based methods have also been proven useful for this purpose [[24], [25], [26], [27], [28]].

The use of novel biomarkers to identify the origin of gelatin represents a significant improvement in the field as these biomarkers offer more precise and reliable methods for gelatin authentication. This data can be utilized to strengthen quality control, guarantee food safety, and shield customers from food fraud [[2], [3], [4], [5]]. Hence, this review defines and highlights the methods currently available to establish the origin of gelatin, including the recent developments in proteomic approaches, including chromatography, mass spectroscopy, electrophoresis, lateral flow devices, and enzyme-linked immunosorbent assay methods, as well as genomic approaches based on nucleic acids and PCR detection, including singleplex PCR, PCR southern hybridization, and multiplex PCR methods. The main advantages and challenges of each of these approaches will also be discussed, along with the future outlook on the field and the main research gaps needed to be addressed for an efficient identification of the original biological materials from which different gelatins are generated. Several databases (PubMed, Scopus, the Web of Science, and Google Scholar) were used to identify the sources of information relevant for this review, including sources dating since 1980 onwards until the most recent developments in the field. The criteria used for the selection of the sources of information as tailored to proteomic and genomic biomarkers and their potential application in the field of gelatin authentication.

## 2 Biomarkers: definition, classification, and applications

Biomarkers are powerful tools used in various sectors by researchers and industries interested in drug development in the pharmaceutical industry, as well as authentication of materials in the food industry. On the other hand, biomarkers can be classified based on various factors, such as their characteristics, applications, and their usefulness when making reasonable and valid decisions. From their characteristics point of view biomarkers are classified as imaging biomarkers (3D tomography, positron emission tomography, X-ray magnetic resonance imaging) and molecular biomarkers (non-imaging biomarkers that have biophysical properties and include nucleic acid molecules, proteins and peptides, lipids metabolites, etc.). Based on their application these biomarkers can be divided into several classes, such as diagnostic biomarkers, staging of disease biomarkers, disease prognosis biomarkers, etc. The last categorizing parameter of biomarkers is the making reasonable and valid decisions for early drug development For example, pharmacodynamic biomarkers are indicators of a specific pharmacological reaction and are particularly relevant to investigations of dosage optimization [[29], [30], [31]].

**Fig. 1:**
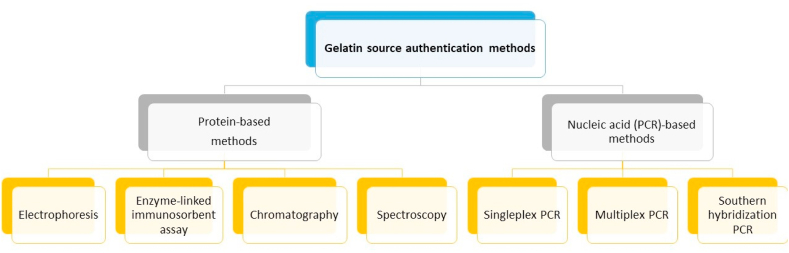
General classification of methods for gelatin source authentication.

The use of biomarkers in the pharmaceutical sector for multiple purposes have been widely researched [[32], [33], [34], [35], [36]]. However, few advances have been made in relation to the use of biomarkers in the food sector. Few research is currently available on the use of biomarkers, such as fatty acids [37], stable isotopic elements [38], proteins and peptides [38,39], and RNA-DNA [[40], [41], [42]], evidencing a research gap worthy of further investigation. One of the most important applications of biomarkers in food science is the authentication of food materials and supplements, and thus, biomarkers for the authentication of the sourcing of gelatin and methods have been researched and they can be classified as shown in Fig. 1.

## 3 Proteomic biomarkers of gelatin

Each method of authentication of gelatin sourcing can have multiple procedures to determine and evaluate different biomarkers. It should also be noted that the application of each method will be highly dependent on the availability of different equipment in laboratories and industries and thus, the main apparatus needed for each method will also be mentioned together with the principles of each approach and the strength of each method for the identification and evaluation of gelatin.

### 3.1 Electrophoresis techniques

Electrophoresis is a valid method used for identification of proteins. This method is based on the migration of proteins through an electric field and the separation of units and subunits based on their charge or isoelectric focusing (IEF) and molecular weight (MW). In case of IEF, the process is performed in a gel, benefiting from the fact that the charge of the molecule of interest will vary as a function of pH [43]. Using a 2-dimensional electrophoresis (2-DE) technique, these two procedures (IEF and MW based) can be combined to separate the proteins components appropriately. Moreover, 2-DE can also be combined with mass spectrometry techniques to improve the discovery of biomarkers [44]. Sodium dodecyl sulfate polyacrylamide gel electrophoresis (SDS-PAGE) is one of the most commonly used methods for the detection of biomarkers of gelatin source authentication [45]. In this method, gelatin with an overall negative charge migrates in an electric field toward anode pole. Gelatins from different origins have various subunits with different MW which can be used to identify their sources. Generally, gelatin subunits are represented in SDS-PAGE gels as α, β, and γ chains with molecular weights of 100, 200, and 300 kDa, respectively. In attempts to represent the fish gelatin as a proper replacement for commercial porcine and bovine gelatin, the SDS-PAGE pattern of these new gelatin has been analysed using this procedure [[46], [47], [48]].

**Fig. 2:**
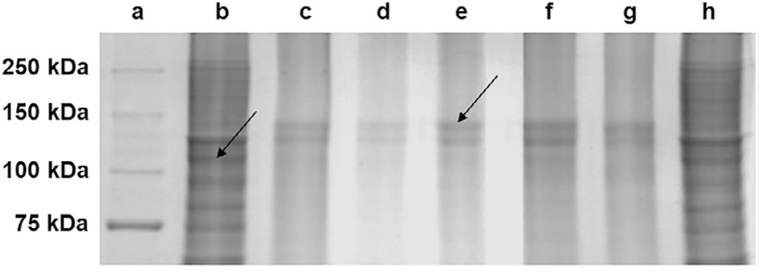
SDS-PAGE pattern of bovine and porcine gelatin. The lanes of the gel are: (a) Protein ladder, (b, h) porcine gelatin standard, (c, d) soft bovine gelatin, (e) bovine gelatin standard, (f, g) hard bovine gelatin. The bands that allowed the differentiation between porcine and bovine gelatin are shown with arrows. Image reproduced from Yap and Gam [48] and reproduced with permission from Elsevier.

Research with the objective of differentiation of the bovine gelatin capsules from the porcine ones, identified gel electrophoresis as a powerful tool during this process [49]. During this study, gelatin proteins were subjected to ammonium sulfate precipitation before SDS-PAGE analysis. 13 double blind gelatin samples were tested and in all cases the method developed was able to show a clear and accurate differentiation between porcine and bovine gelatin samples, with distinctive protein bands at 110 and 140 kDa for porcine and bovine gelatins, respectively (Fig. 2a–h). These 2 protein bands could be used as biomarkers for the differentiation between these 2 gelatins; however, further investigation and characterization of these bands with other techniques such as enzymatic hydrolysis and finding their amino acid sequences with tandem mass spectroscopy will be needed in order to advance knowledge on these biomarkers.

Some of the main advantages of the SDS-PAGE are the cost effectiveness and availability of the apparatus that can be present in most laboratories as they are economical, convenient, and versatile in application [50,51]. Moreover, the method is relatively simple to perform and fast even when it is used in combination with other techniques [51,52]. However, this method also has certain disadvantages, such as the high probability of laboratory errors and the high toxicity of certain materials and reagents used, like acrylamide [[53], [54], [55], [56]].

### 3.2 Enzyme-linked immunosorbent assay (ELISA)

ELISA is a technique based on the detection of an immunological response resulting from a molecule generated during specific antibody-antigen reactions [57]. It should be mentioned that this method is capable of detecting a wide variety of biomarkers either proteomic or genomic [58]. This method has been widely used for gelatin authentication purposes (see Table 1). Although the general principles of this method were mentioned previously, there are 4 types of ELISA that can be used for gelatin sourcing authentication. All these methods and their specifics are discussed below, and a concise summary of the different ELISA methods and their advantages and limitations are listed in Table 2.

**Table 1:** Advantages and limitations of different ELISA methods.

Method	Advantages	Limitations
Direct ELISA	● Cheap ● Simple ● Elimination of cross reactivity of secondary antibody	● Minimal signal amplification ● Less specificity ● The primary antibody has to be individually labelled, which is time-consuming and expensive ● Primary antibody label selection is inflexible from one experiment to another.
Indirect ELISA	● Flexible to use variety of antibodies ● Simple ● Elimination of cross reactivity of secondary antibody	● Cross-reactivity might occur with the secondary antibody, resulting in non-specific signal ● Time consuming and more expensive
Competitive ELISA	● High sensitivity ● Suitable for complex samples ● Flexibility	● Difficulties to find another protein that be recognized by primary antibody
Sandwich ELISA	● High specificity ● Suitable for impure samples	● False positive results

The information in this table was summarised from multiple scientific publications [58,59].

**Table 2:** Application of ELISA methods in gelatin source authentication.

ELISA type	Antibodies used	Targeted species	Main results	References
Indirect and competitive indirect	Polyclonal anti-peptide antibodies	Distinguishing porcine from bovine gelatins	For discovering bovine gelatin present in porcine gelatin samples to a dilution of 2–4 parts per 1000, competitive indirect ELISA proved more suitable.	[60]
Sandwich ELISA	Polyclonal antibodies from immunization of gelatin in rabbit (pAb2-pAb1) and in goat (pAb3-pAb3)	Identifying porcine and bovine gelatins in food processing for individuals at risk of gelatin allergy	Porcine gelatin (alkaline process) was the primary target of the pAb2-pAb1 ELISA technique whereas bovine and porcine gelatin were the primary targets of the pAb3-pAb3 ELISA (alkaline process). All commercial products that were stated to include gelatin were successfully detected by both ELISA methods.	[61]
Indirect ELISA	Anti-peptide polyclonal antibodies	Analyzing the porcine gelatin content of an edible bird's nest	The established ELISA method detected spiked samples containing at least 0.5 ng/μg porcine gelatin.	[62]
Indirect ELISA	Anti-peptide (collagen α2) polyclonal antibodies	Verification of the source of the gelatin in confectionary products	The detection limit of the specially designed ELISA was 0.05 μg/mL, and it demonstrated little cross-reactivity to both fish and chicken gelatin.	[63]
Indirect ELISA	Anti-peptide polyclonal antibodies	Analyzing the porcine gelatin content of an edible bird's nest	The sequence using collagen α1 was discovered to be sufficient for authenticating edible bird's nest with a detection limit of 0.052 g/mL among three porcine species-specific peptides.	[64]

#### 3.2.1 Direct ELISA

**Fig. 3:**
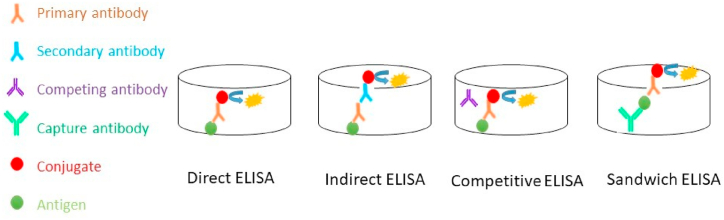
A schematic representation of the principles underlying different ELISA procedures.

This assay comprises 2 steps as represented in Fig. 3. The first step is the introduction of the sample of interest in the surface of the wells in ELISA microplates followed by the addition of an enzyme-labelled primary antibody. The substrate and stopping agents are then added to the wells and the signal is then recorded. This protocol is so easy to perform and has some limitations, mainly less specificity, time-consuming and expensiveness of labeling and inflexibility amongst others as summarised in Table 2. To our knowledge there is no research using direct ELISA for the authentication of the sources of gelatin. However, this procedure coupled with other verification methods, such as electrophoresis or nanotechnology, used for the detection of cancer biomarkers [59] could also be applied to identify the sources of gelatin.

#### 3.2.2 Indirect ELISA

Indirect ELISA has been extensively used for the authentication of the sources of gelatin [60,61]. This protocol is based on immobilizing the antigens (gelatin samples) onto the surface of microplate wells an adding a primary antibody that in this case will attach to the samples. Secondary antibodies labelled with a conjugate are then added to attach to the primary antibodies [62]. The main reason for the widespread application of this method for gelatin authentication is its flexibility and wide variety of antibodies available to be used in these tests. The antibodies exhibit significant variations in their sensitivity to species and production method when tested by indirect ELISA. Some antibodies are quite sensitive to the acid or alkaline processing of bovine and porcine gelatins, and some can indicate the presence of gelatin in food [63].

#### 3.2.3 Competitive ELISA

In competitive ELISA, the primary antibody is competitively attached to the sample antigen and the secondary antibody coupled to the detection enzyme binds to the primary antibody [64] as seen in Fig. 3. This protocol is commonly applied when the antigens are small and with only one antibody-binding site [65]. The main advantages and limitations of this method are listed in Table 2. This method sensitive and suitable for complex samples containing gelatin. Although this method could be used for the authentication of the origin of gelatin, several conditions used for the production of gelatin, including the alkaline/acidic pre-treatments and the type of by-products (skin, bone, and hide) present can negatively affect its sensitivity [61,63]. For instance, the fish gelatin could be obtained from different by-products, such as skin [66], scales [67], and even a combination of them [48]. To overcome this problem, an indirect competitive ELISA method was examined using a polyclonal antibody produced utilizing maleimide activated keyhole limpet hemocyanin coupled with peptides from rabbit. This reagent could raise antibodies against specific peptides present in porcine collagen α1 (I) chain pAb3, α2 (I) chain pAb1, and pAb2, with 14–22 amino acid residues. pAb3 peptides could be used as a tool to originate gelatin with high levels of accuracy, specificity, and repeatability [48]. However, it should be noted that this procedure will increase the cost of the laboratory analyses and that the accessibility of the specific reagents used in this method could limit its widespread application.

#### 3.2.4 Sandwich ELISA

In a sandwich ELISA, 2 antibodies attach to the sample antigen and one of them is coupled with the conjugate (Fig. 3). In an attempt to use sandwich ELISA to authenticate the origin of the gelatin samples, Doi, Watanabe [68] used 2 polyclonal antibodies obtained from rabbit and goat. The results of this study revealed that the sandwich ELISA can be used successfully for determination of the porcine and bovine gelatin. However, the authors also appreciated a reactivity of the antibodies with fish gelatin, limiting the applications of this method as it cannot discriminate in a reliable manner between gelatins from different sources [68]. Moreover, the authors stated that another limitation of this method was obtaining false responses after gelatinization or as a result of acidic pre-treatment [68].

### 3.3 Chromatographic techniques

Chromatography is an easy, rapid, and economical technique which has been used successfully for purification and identification of compounds [[69], [70], [71]]. There are 2 general categories of this method, based on the type of eluent, namely gas chromatography that uses gas as an eluent and liquid chromatography, in which the eluent is in liquid form.

#### 3.3.1 Gas chromatography (GC)

Gas chromatography (GC) is a sensitive and powerful technique that can be used for the simultaneous detection and separation of volatile or semi-volatile target molecules [72,73]. However, there are some limitations to GC, such as the requirement of the analytes to be volatile and the need of several pre-separation and concentration steps of the compounds from the food matrix before analysis that can lead to contamination and sample loss [[73], [74], [75]]. To the best of our knowledge, no research has been conducted to date exploring the capability of GC for the authentication of the sources of gelatin. However, this method could have the potential to discriminate the gelatin samples based on their fat contents which means it could be used to detect fatty acid biomarkers in gelatin samples obtained from different sources.

#### 3.3.2 Liquid chromatography

Liquid chromatographic methods are based on 3 principal chemical properties including size of the molecule, electrical charge, and polarity [76]. These techniques have been used successfully in combination with other methods, such as mass spectroscopy, to increase their sensitivity and achieve more reliable responses for the purposes of authentication of the sources of gelatin (see Table 3) [77]. Among different liquid chromatography methods, the reverse phase high performance liquid chromatography (RP-HPLC) has been extensively used for the separation and authentication of biological molecules with similar structures, functions, and behaviour. RP-HPLC method provides reliable results for a wide range of biological macromolecules. This method, especially when used with fluorimeter can be a very sensitive technique [76,78]. In this regard, Widyaninggar, Triyana [79] designed an experiment and demonstrated how RP-HPLC method can distinguish between species-specific gelatin products. The authors used an acidic hydrolysis procedure to determine the amino acid profiles of the capsule shells comprising gelatin. A principal component analysis (PCA) score plot was used to distinguish the differences in the amino acid profiles of the two gelatin sources, and the principal components (PC) PC1 and PC2 showed 64.4% and 15.7%, respectively, within the bovine and porcine samples. In another study, RP-HPLC was used to identify sixteen amino acids distinguishing the gelatin sources. The original data matrix was changed into a number of principle components through PCA. The PCA model illustrated the connections between the group of gelatins in the scores plot and the correlation loadings plot between amino acids. Threonine, serine, and methionine were correlated with fish gelatin on the positive side of PC1, while proline, hydroxyproline, leucine, isoleucine, and valine were correlated with bovine gelatin on the negative side of PC1, and aspartate, glutamic acid, lysine, and tyrosine were correlated with porcine gelatin on the negative side of PC2 [80].

**Table 3:** Application of chromatographic techniques in gelatin source authentication.

Chromatographic techniques/detectors	Targeted biomarkers	Chromatographic conditions	Main results/limitations	References
RP-HPLC-MS/MS	Peptides (STGISVPGPMGPSGPR and SAGISVPGPMGPSGPR)	Mobile phase of HPLC was water and acetonitrile containing acetic acid. MS analysis was conducted with mass scan range of 400–900 m/z	Possible to distinguish between bovine and porcine gelatins by identifying the marker peptides in the digested gelatins using HPLC/MS	[83]
RP-HPLC-MS/MS	Peptides (GPPGSAGSPGK and GPPGSAGAPGK)	Mobile phase of HPLC was water and acetonitrile. MS analysis was conducted with mass scan range of 400–900 m/z	HPLC-MS/MS identified peptides in the digested gelatin sample able to distinguish between porcine and bovine gelatin. The sequence should be checked and verified for peptides like GPPGSAGSPGK and GPPGSAGAPGK seen in digested bovine and porcine gelatin, as the mass shift brought on by proline hydroxylation might be misinterpreted with the mass difference of Serine and Alanine residues	[84]
RP-HPLC-Fluorescence	Aspartic acid, Hystidine, Phenylalanine, Isoleusine, Lysine Glutamic acid, Glycine, Threonine, and Tyrosine	Mobile phase was acetate buffer (pH 5.9) a mixture of methanol: Acetate buffer: tetrahydrofuran (400:75:25 v/v). The peak regions at 340 nm excitation and 450 nm emissions were measured in order to quantify the amino acid profiles	Gelatin products made from different species can be distinguished using the RP-HPLC technique	[81]
RP-HPLC-Fluorescence	Threonine, Serine, Methionine, Proline, Hydroxyproline, Leucine, Isoleucine, Valine, Aspartate, Glutamic acid, Lysine, and Tyrosine	AccQ.Tag™ Eluent A, concentrate (WAT052890), deionized water, and acetonitrile were utilized as the system's tertiary solvents. The peak regions at 250 nm excitation and 395 nm emissions were measured in order to quantify the amino acid markers	The grouping patterns and variable correlations were verified on the database using 12 samples from commercial gelatin-based products. In order to ascertain gelatin from diverse sources, this quantitative approach is therefore highly helpful as a screening tool	[82]
HPLC-UV	Asparagine and Glutamine	Mobile phase was 40 mM acetate buffer (pH 5.5) along with methanol. The peak regions at 330 nm wavelength were measured in order to quantify the amino acid markers in pulsed electric field and OPA pretreated samples	Animal-derived gelatin could be identified using O-phthalaldehyde derivatized amino acids assessed by the pulsed electric field and HPLC-UV assays	[85]
RP-HPLC-MS/MS	Peptides (QGPSGPSGER, GETGPAGPAGPVGPVGAR and SAGISVPGPMGPSGPR)	HPLC mobile phase was water and acetonitrile containing formic acid. MS analysis was conducted with mass scan range of 300–1800 m/z.	The reliable authentication of porcine gelatin can benefit greatly from these frequent characteristic tryptic peptides	[86]
UPLC-MS/MS	Peptides (AGVMGPOGSR, GETGPAGPAGPVGPVGAR, and GEOGPTGVQGPOGPAGEEGK)	HPLC mobile phase was water and acetonitrile containing formic acid. MS analysis was conducted with mass scan range of 200–1500 m/z.	The presence of porcine gelatin could be efficiently determined at a level of 0.04%.	[21]
UPLC-MS/MS	Peptides (GNDGATGAAGPHypGPTGPAGPHypGFHypGAVGAK, GSDGSVGPVGPAGPIGSAGPHypGFHypGAHypGPK, GSDGSVGPVGPAGPIGSAGPHypGFPGAHypGPK, GFHypGTHypGLHypGFK, and GNDGATGAAGPHypGPTGPAGPHypGFPGAVGAK)	HPLC mobile phase was water and acetonitrile containing formic acid. Analysis was conducted with mass scan range of 350–1800 m/z.	The biomarkers could be used for distinguishing the deer horn gelatin and hide gelatin, as well as calculating adulteration in commercial gelatins.	[87]

The HPLC methods have been used to monitor the amino acid profile in gelatin samples, achieving distinctive chromatograms and the peak heights that can be useful when differentiating gelatin samples of different origin. The amino acid content of gelatins with different origins can be detected using HPLC with precolumn derivatization [79,80]. Porcine gelatin consists mainly of 2 amino acids (asparagine and glutamine) that can be used as a differentiating parameter to distinguish porcine from bovine gelatins that are deficient in these amino acids. However, due to their hydrophilicity and poor UV absorption, these two amino acids are difficult to identify [77,80]. Rezazadeh, Yamini [81] developed a practical approach aiming to solve the aforementioned issues. O-phthalaldehyde was used to derivatize amino acids from a few specific species in order to boost their hydrophobicity and UV absorbance. The authors used a 137 V electric field with a 10 min^−1^ frequency for 20 min to move the analytes across a 200 μm organic liquid membrane into an aqueous acceptor phrase. An HPLC system with UV detector was used to evaluate the aqueous phase at the conclusion of their experiment. The limit of detection for asparagine and glutamine were 25 and 50 ng/mL, respectively, whereas the assay had extraction recoveries of 43% and 79%.

The main challenge associated with chromatography methods is that it requires many chemometric or mathematical analyses to properly authenticate the origin of different gelatins. More recent techniques, such as coupling mass spectrometric methods and using bioinformatics tools to match the obtained data with database, have contributed greatly to solve these issues and ease the procedures.

### 3.4 Spectroscopy

#### 3.4.1 Mass spectrometry (MS)

MS technique is frequently coupled with liquid chromatography or used as the identification tool after fractionation and purification by different liquid chromatographic methods. MS can be applied to analyse gelatin by the molecular weight distribution and amino acid sequence of the molecules. Proteomic approaches using MS possess the advantage of being strong and solid, and trustworthy for gelatin biomarkers identification (See Table 3).

**Fig. 4:**
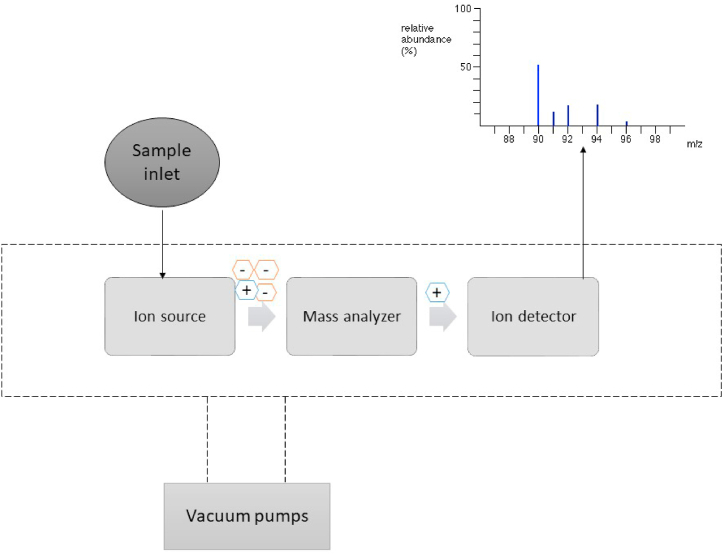
Schematic view of mass spectroscopy.

MS is an analytical technique working based on measuring the mass of the samples. The most general types of MS are the electrospray ionization and matrix-assisted laser desorption/ionization. The advantage of these ionizers in MS techniques is that the molecules in the gelatin sample remain fairly intact during the ionization [82]. Generally, MS includes three major parts, the ionizer, mass analyser, and detector, connected to a computer. Samples (gelatin hydrolysates obtained usually from enzymatic hydrolysis reaction) are first ionised getting positive charges. These ions will be sorted and deflected based on their mass and charge ratio (*m*/*z*) in the mass analyser. Subsequently, the detector plates will multiply the ions to enhance sensitivity. The spectra will be plotted in the computer as *m*/*z* against relative abundance and checked through databases (bioinformatics). These procedures are summarised schematically in Fig. 4. MS with two mass analyzers or more are known as tandem mass spectrometry and these apparatus could be useful when identifying peptide biomarkers [82]. Recently, high-performance liquid chromatography tandem mass spectrometry (HPLC-MS/MS) approaches serve as the basis of proteomics and peptidomic studies. This method has been extensively suggested to determine the source and monitor the quality of proteins as a result of their significant specificity and sensitivity in discovering species-specific biomarkers [21,22,83,84]. Liu and Huang [21] developed a method for identifying the various gelatin sources using peptide biomarkers [21], and a process was developed for measuring the level of adulteration of gelatins using nanoLC-MS/MS that boosted the MS signal [85]. Moreover, other reports specify that the addition of small amounts of glycine may also improve the responses and detection of peptides when using LC-MS/MS based characterisation of biopharmaceuticals [86].

#### 3.4.2 Infrared spectroscopy (IR)

**Fig. 5:**
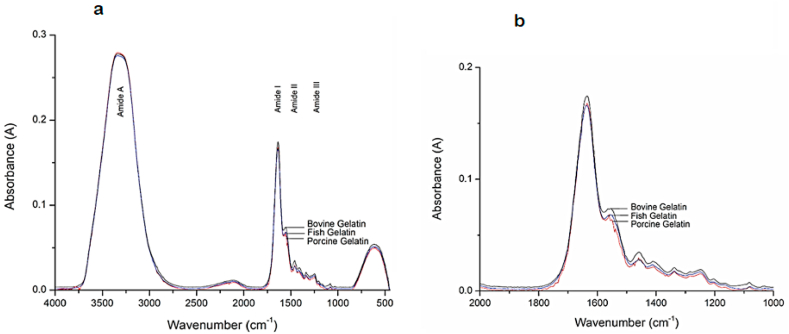
FTIR spectra used to differentiate the gelatin sources of bovine, porcine, and fish. (a) Full scale FTIR spectra of bovine, porcine and fish gelatins and (b) enlarged spectra of bovine, porcine and fish gelatins at 2000–1000 cm^−1^. Figure originally published in MDPI by Hassan, Ahmad [89].

IR spectroscopy has been used in a huge range of applications from the analysis of molecules to cells or tissues [87]. FTIR concerns with the examination of proteins to scan their conformation, folding, and molecular details [88,89]. This technique can be used to compare the spectra achieved from different species and differentiate them (see Table 4). The full scale and enlarged spectra of bovine, porcine, and fish sources of gelatin are shown in Fig. 5a and b in which significant peaks are indicated in Amide A, I, II, and III regions at wavenumbers of 3310–3270 cm^−1^, 1700-1600 cm^−1^, 1550-1400 cm^−1^, and 1240-670 cm^−1^, respectively [90]. These spectra were subjected to chemometrics fuzzy autocatalytic set analysis, and the results revealed that each gelatin had dominant wavenumbers at 1470-1475 cm^−1^, 1444–1450 cm^−1^, and 1496–1500 cm^−1^, respectively, which serve as their distinctive signatures.

**Table 4:** Application of IR techniques in gelatin source authentication.

IR technique	Targeted species	Main results/limitations	References
**FTIR- ATR**	Bovine and porcine	The unknown origin of gelatin could be identified using discriminant analysis for N–H bond deformation in the ranges of 3290 to 3280 cm^−1^ and 1660 to 1200 cm^−1^	[96]
**FTIR**	Bovine and porcine	On both sources of gelatin, variations were seen in the range of 2800–3000 cm^−1^, which denotes the presence of an aliphatic C–H stretching area, and in the region of 1543 cm^−1^, which demonstrated a C–N–H bending of the peptide bonds. The range of 1450–1300 cm^−1^ (*C*–H bending), which indicates variations in the amino acid composition of the two sources of gelatin, was the third difference	[97]
**FTIR-ATR**	Fish, bovine, and porcine	Compared to other gelatin species, fish gelatin has distinct spectral characteristics in the 1100-1000 cm^−1^ spectrum region that are associated with the *C*–O stretching vibrations of the carbohydrate residues	[98]
FTIR- cFACS*	Fish, bovine, and porcine	The major wavenumbers for bovine, porcine, and fish gelatins were determined to be 1475–1470 cm1, 1450–144 cm^−1^, and 1500–1496 cm^−1^, respectively. These wavenumbers were related to N–H bond deformation in the Amide II region	[99]
**FTIR- cFACS**	Fish, bovine, and porcine	The main distinctive wavenumbers for bovine, porcine, and fish were detected at 1470–1475 cm^−1^, 1444–150 cm^−1^, and 1496–1500 cm^−1^, respectively. These wavenumbers are related to the deformation of N–H bonds in the Amide II region	[95]

* Fourier transform infrared spectroscopy in combination with chemometrics fuzzy autocatalytic set.

On the other hand, IR spectroscopy or FTIR is also could be used to originate the sources of gelatin using biomolecular fingerprinting, such as identification of lard (pig fat) [[91], [92], [93]]. These studies revealed that slight differences in the fingerprint regions of FTIR spectra could be used to detect pig fat or its other derivatives. This peak observation is the tool and minor changes in the spectra could be interpreted as the biomarker for gelatin originating. Another way of application is the analysis of the hydrolysed gelatin samples from different origins. The minor differences between amino acid compositions of bovine and porcine gelatins (glycine, proline, and arginine at the range of 2800–3000 cm^−1^, 1543 cm^−1^, and 1450 to 1300 cm^−1^) could be detected by FTIR spectra, which means these amino acids at the especial wavenumbers could be used as biomarkers to determine the source of the gelatin samples [94]. Despite the fact that the FTIR is a strong method to discriminate gelatin sources, computing principle component analysis and chemometric tools are needed to establish an accurate and precise method. Hashim, Man [95] developed a straightforward and swift procedure using FTIR in combination with attenuated total reflectance (ATR) and discriminant analysis to differentiate the sources of gelatins. This analysis provided a distinctive variation between bovine and porcine gelatins in the regions of 3290–3280 cm^−1^ and 1660–1200 cm^−1^ [95].

IR spectroscopy is a powerful tool that could be strengthen further by other spectroscopic techniques, such as fluorescence spectroscopy that has been proven as a mighty tool to analyse the structure of molecules. The combination of these tools could help to increase the precision of the identification of the sources of origin of gelatin. Zhang, Liu [96] reported that the origin of gelatin could be predicted based on data available from near infrared spectroscopy (NIRS), fluorescence spectroscopy, and laser-induced breakdown spectroscopy (LIBS). The accuracy of single techniques and the data fusion strategy were determined to be 97.1%, 98.55%, 81.16%, and 100%, respectively.

## 4 Genomic biomarkers

Genomic biomarkers used for the purpose of authentication of the sources of gelatin are based on the polymerase chain reaction (PCR), a powerful technique to detect the presence and determine the quantity of DNA [27]. This method is used for the identification of DNA molecules of different origins (i.e. pork, bovine and chicken) with different methods including southern-hybridization, and Singleplex PCR [25,77].

YOGI [97] and Sahilah, Liyana [98] developed a PCR procedure to detect pork DNA in gelatinous capsules based on southern-hybridization. This method has been promisingly applicable to detect pork DNA in meat and surimi products [99].

Real-time PCR, also known as quantitative PCR, is a method based on the PCR by which an amplification of a targeted DNA is monitored. Real-time PCR is currently used to detect the presence of undesirable DNA in different food and pharmaceutical products [17,28,100,101].

**Fig. 6:**
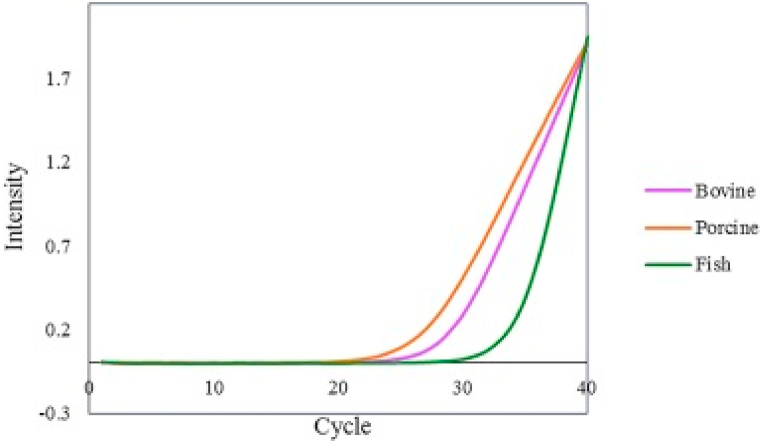
Real time PCR amplification of DNA extracted from gelatin by different specific primers for bovine, porcine and fish. Figure originally published in ELSEVIER by Jannat, Ghorbani [27].

The use of real time PCR for the authentication of the sources of gelatin is based on the detection of DNA in gelatin samples determined by the cycle threshold (Ct) value (the number of cycles needed for the fluorescent signal to reach the threshold) and amplification curve [102]. In gelatin authentication, the least quantity that might be amplified with a reproducible Ct value is known as the detection limit [103]. This method is an effective tool to identify the source of gelatin; however, in some cases a misdetection of amplification signals can happen as a result of possible DNA denaturation [28]. Fig. 6 indicates the real time PCR amplification curve of DNA extracted from gelatin by different specific primers for bovine, porcine and fish.

**Fig. 7:**
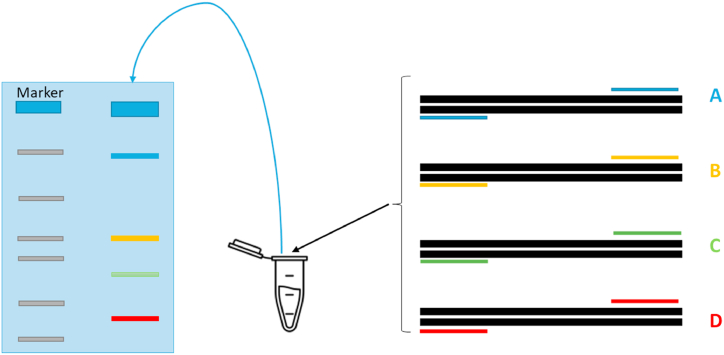
Schematic representation of multiplex PCR.

Other approaches based on DNA beside PCR include PCR-restriction fragment length polymorphism (RFLP), species-specific PCR, and multiplex PCR [25,27,[104], [105], [106], [107]]. After the DNA samples in concern have been digested with the appropriate restriction endonucleases, the presence of fragments of various lengths can be used to identify differences in homologous DNA sequences, or RFLP. As a molecular marker, RFLP is unique to one particular clone/restriction enzyme pair. An RFLP probe is a labelled DNA sequence that, after being separated by gel electrophoresis from one or more digested DNA sample fragments, hybridizes with those fragments to display a distinctive blotting pattern specific to a particular genotype at a particular locus [108]. The approach that uses species-specific primers is known as specific-specific PCR. The concept of PCR primer sets that amplify DNA from just one species is the basis for the idea of species-specific primers [107]. Multiplex PCR involves the use of two or more primer sets for the simultaneous amplification of several targets (Fig. 7). Several target sequences in a sample can be amplified in a single vial using this method [109].

The primers used in PCR are reliable biomarkers for gelatin origin authentication and allow the discrimination between pork, bovine, chicken, and fish gelatins. Table 5 summarises the main primers related to different sources of gelatin that have been proven successfully to determine the original sources of gelatin and can be used as biomarkers to discriminate them. Nevertheless, due to the tremendous number of fish species (over 30,000), the determination of fish origin in samples containing fish gelatin with development of specific primers for each individual species is particularly challenging [110]. This challenge could be overcome by generation of a pair of universal primers for fish [25] (see Table 5).

**Table 5:** Primer biomarkers applicable in gelatin source authentication.

Species (origin)	Sequence (5′–3′)	Size (bp)	Target genes	References
Porcine	Forward: ATTTCCATCCCACAGCCC Reverse: AACAGATGCTGACTCACAGAC	–	MPRE42	[118]
Bovine	Forward: GCCTAAATCTCCCCTCAATGGTA Reverse: ATGAAAGAGGCAAATAGATTTTCG	271	Cyt *b*	[119]
Porcine	Forward: GACCTCCCAGCTCCATCAAACATCTCATCTTGATGAAA Reverse: GCT GAT AGT AGA TTT GTG ATG ACC GTA	398	Cyt *b*	[120]
Bovine	Forward: CGGCACAAATTTAGTCGAAT Reverse: TGGACTATGGCAATTGCTATG	120	Cyt *b*	[121]
Universal fish primer	Forward: ATCACAAAGACATTGGCACCCT Reverse: AATGAAGGGGGGAGGAGTCAGAA	295	COI	[24]
Porcine	Forward: TTACGGATGAGTTATTCGCTACCTA Reverse: TATAGGCCTCGCCCTACGT	87	Cyt *b*	[24]

Despite the fact that the genomic biomarkers determination is a precise and efficient way to demonstrate the origin of gelatin, several parameters including the high cost of equipment and reagents for PCR analysis, low stability of the DNA, need for large amount of pure DNA, and time needed to perform this technique (up to 6 h depending on the sample) are strong factors that limit the widespread use of this technique for the purpose of gelatin source authentication [[111], [112], [113]].

## 5 Conclusions and future outlook

Two prominent approaches for the identification and authentication of the origin of gelatins based on proteomic and genomic biomarkers have been explored. Some approaches based on combination of different analytical methods, such as microfluidics and microchip electrophoresis techniques, have been successfully used for the detection of clinical biomarkers so far. On the other hand, when using 2-DE technique, two important factors of IEF and MW can be combined to separate the proteins components appropriately. Moreover, 2-DE can also be combined with mass spectrometry techniques to improve the discovery of biomarkers. ELISA, a method that has been widely used for gelatin authentication purposes, could be applied in four different ways, including direct, indirect, sandwich, and competitive ELISA. Each of them has advantages and limitations in its application. Moreover, IR spectroscopy in combination with chemometric methods has also been reported as a powerful and reliable technique for gelatin authentication. The precision of this method could be enhanced by other spectroscopic techniques, such as fluorescence spectroscopy that has been proven as a mighty tool to analyse the structure of molecules. Further research is still required to fully understand how effective NIRS can be as a method for gelatin authentication. Amongst the spectroscopic methods, the use of chromatography and mass spectrometry, is another example of how coupling analytical techniques can be a useful strategy when establishing proteomic methods as the combined detection method offers better results compared to the original protocols when applied individually. As per the time of this study, several specific PCR assays are considered as effective methods for the determination of the origin and authentication of gelatin of bovine, porcine, fish, and plant origin used as dietary supplements. PCR, especially when coupled with mass spectroscopy, could be a powerful approach for gelatin source authentication in the future. Another technique that is gaining momentum is Raman spectroscopy, as it is a non-destructive method with high sensitivity and specificity. However, at the present time there is currently not enough research performed evaluating this method for the purpose of gelatin authentication. However, and as seen in previously explored techniques, the use of Raman spectroscopy alone or combined with other techniques could have multiple advantages worth of being explored in future research developing a powerful tool for the authentication of the source of gelatin, as it has been proven that this procedure can be adapted for protein and DNA biomarker analyses.

Altogether, even though using biomarkers to identify the origin of gelatin has shown potential, there are still a number of research gaps that need to be elucidated. The development of thorough and well-established biomarker panels, the confirmation of biomarkers for a broader variety of species, a better comprehension of the constraints and possible uses of the methods of detection, and the affordability of their application are a few of these issues that are yet to be addressed.

## Author contribution statement

All authors listed have significantly contributed to the development and the writing of this article.

## Data availability statement

Data included in article/supplementary material/referenced in article.

## Additional information

No additional information is available for this paper.

## Funding

Dr. Armin Mirzapour-Kouhdasht works within the project AMBROSIA (code: 2020HDHL102) funded within the 10.13039/501100007601Horizon 2020 Joint Programming Initiative (ERA HDHL PREVNUT 2020) project administered by the Department of Agriculture Food and the Marine (DAFM).

## Declaration of competing interest

The authors declare that they have no known competing financial interests or personal relationships that could have appeared to influence the work reported in this paper.
